# Exclusive breastfeeding knowledge and practice among nursing mothers in selected healthcare facilities in Kaduna Metropolis, Nigeria

**DOI:** 10.4314/ahs.v23i2.78

**Published:** 2023-06

**Authors:** Musa I Yakubu, Rachael U Odesanya, Medinat Y Abbas, Basira K Lawal

**Affiliations:** 1 Department of Pharmacology and Toxicology, Kaduna state University, Kaduna, Nigeria; 2 Department of pharmacy, Jos University Teaching Hospital, Plateau state, Nigeria; 3 Department of Pharmacology and Therapeutics, Ahmadu Bello University, Zaria, Nigeria; 4 Department of Clinical Pharmacy and Pharmacy management, Kaduna state University, Kaduna, Nigeria

**Keywords:** Exclusive breastfeeding, knowledge, initiation of breastfeeding, nursing mothers

## Abstract

**Background:**

Exclusive breast feeding (EBF) in the first six months of life is recognized as an indispensable component of survival, physical and mental development of children. Despite the enormous benefits of EBF, only 39% of infants less than 6 months of age are exclusively breastfed globally.

**Objectives:**

This study assessed EBF knowledge, practice and associated factors among nursing mothers attending health facilities in Kaduna metropolis.

**Methods:**

Descriptive and cross-sectional study carried out with the aid of a structured questionnaire at the Barau Dikko Teaching Hospital and Gwamna Awwal General Hospital in Kaduna. The participants were recruited by simple random sampling technique with the assistance of trained research assistants for four consecutive weeks in each of the hospital. Data were analysed using SPSS version 16.

**Results:**

Majority of the participants were in the age range of 21-30 years. Their main source of information on breastfeeding was from ante-natal clinics (78.3%). Most (86.6%) of the participants had a good knowledge and awareness about EBF, 69.1% of them initiated breastfeeding immediately after birth (within an hour) and 70% of the participants practiced EBF. Insufficient milk, belief that infants require extra fluid, fear of alteration in the breast figure, cultural practice and societal beliefs were identified as the most common barriers to the practice of EBF.

**Conclusions:**

Findings from this study showed that most of the participants had a very good knowledge of EBF and are practicing it.

## Introduction

Exclusive breastfeeding (EBF) is the feeding of the infant with only breast milk for a period of 6 months without any additional food or drink, not even water. It provides adequate nutrition required for optimal growth and development of infants in the first six months of life 1. Thereafter, infants should receive adequate complementary foods with continued breastfeeding up to 2 years of age or beyond 2. The World Health Organization (WHO) recommended initiation of breastfeeding within one hour of birth, exclusive breast feeding for the first six months of life and continued breastfeeding up to two years of age or beyond 3. The importance of breastfeeding as a determinant of infant nutrition, child mortality and morbidity has long been recognized and documented in the public health literature 4. EBF practice is strongly linked with a significant reduction in burden of infant and child morbidity and mortality especially among children under five years of age 5.

Despite the enormous benefits of EBF practice, millions of children in many countries of the world are still not exclusively breastfed in the first 6 months of life as recommended by WHO. Globally, only 39% of infants are exclusively breastfed 6. In sub-Saharan Africa, the prevalence of exclusive breastfeeding practice ranges from 23.7% to 56.5% 7. This contributes to the disproportionate burden of under-five child morbidity and mortality in sub-Saharan Africa 8. According to 2018 NDHS 9, the percentage of exclusively breastfed infants in Nigeria has risen from 17% to 29%. The low adherence to EBF practice in Nigeria is associated with diarrhea-related deaths among children younger than five years of age 10. This is inimical to the realization of the Sustainable Development Goal (SDG) target of reducing infant and child mortality rate to a significantly low level by 2030. Most studies on EBF practice in Nigeria were conducted at national and regional levels, but there is limited information on the knowledge and practice of EBF among nursing mothers in Kaduna state. This study is therefore, aimed at assessing the practice of EBF and its associated factors among nursing mothers attending health facilities in Kaduna metropolis, Northwest Nigeria.

## Methods

### Study setting and design

A cross-sectional study was conducted in Kaduna metropolis at Barau Dikko Teaching Hospital in Kaduna North and Gwamna Awwal General Hospital Kakuri in Kaduna South Local government areas of the state. The study population consisted of nursing mothers of newborns and babies less than two years old, attending postnatal clinics of government health facilities in Kaduna metropolis.

### Sample size and Sampling technique

The sample size for the study was determined using the formula; n = Z^2^P (100-P)/X^2^ 11, where n is sample size, X^2^ is level of precision which is 5 %,

Z^2^ is the confidence interval; taken as 95% degree of probability which is 1.96 approximated to 2 and, P is the percentage of women practicing exclusive breastfeeding in Nigeria, which is 17% 12.

100-P is the percentage of women not practicing exclusive breastfeeding.



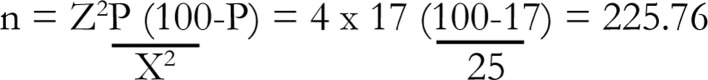



Assuming anticipated 85% response rate and to compensate for attrition, the sample size used was adjusted as follows:


$$\begin{array}{l} 225.76=265\\ \bar{\;\;\;0.85\;\;\;\;} \end{array}$$

The sample size used was 265.

The participants were recruited by simple random sampling technique with the assistance of trained research assistants for four consecutive weeks in each of the health facilities.

Inclusion criteria are mothers who delivered normally, had full term babies weighing ≥ 2.5kg, and the babies less than two years old. Exclusion criteria are mothers who delivered through CS.

### Ethical consideration

Ethical approval (approval no: MOH/ADM/744/VOL.1/33) for the study was obtained from Kaduna state Health Research Ethics committee (HREC) and inform consent was obtained verbally from all the participants.

### Data collection

Structured and validated questionnaire were used to collect data. The questionnaire was designed to elicit information on socio-demographic characteristics, economic status, knowledge and practice of exclusive breastfeeding as well as the constraints to practice of EBF in Kaduna metropolis. The literate nursing mothers were given the questionnaire to fill while the illiterate ones were interviewed from the questionnaires and the answers recorded. The questionnaire was pilot-tested using nursing mothers attending immunization in one of the public health centres within Kaduna metropolis. The questionnaires for the study were sorted for completeness after data collection and incomplete questionnaires were discarded.

The participants' level of knowledge and practice of EBF was assessed as either good, moderate or poor based on: (a) correct definition of EBF and understanding that it entails feeding an infant with only breast milk (except medications) for the first 6 months of life; (b) Timely initiation of breastfeeding (within an hour after delivery); and (c) understanding the benefits of EBF to infants and their mothers.

### Data Analysis

Data collected were analysed using statistical package for social sciences (SPSS) version 16. Descriptive statistics such as mean, standard error of mean, frequencies and percentages were used to analyse data on socio-demographic characteristics of the participants. Chi square test was used to determine the association between some socio-demographic characteristics of the participants and practice of exclusive breastfeeding. Statistical significance was set at 95% confidence interval (p ≤0.05).

## Results

### Socio-demographic characteristics of the participants

Two hundred and thirty nursing mothers (n= 230) completed the questionnaire making the response rate to be 86.8% and Participants ranged in age from less than 20 to greater than 50 years. Participants less than 20 years in age represented 9.1%, those within the ages of 20 – 30 constituted 54.8%, those between the ages of 31 – 40 were 34.8% while those within the ages of 41 – 50 represented 1.3% of the participants. 95.6% of the participants were married, 3.0% were singled and 1.3% separated/divorced. 1.3% of the participants had no formal education, 5.7% had only primary education, 49.1% had secondary education while 43.9% of them had tertiary education. 17.4% of the participants were full house wife, 48.7% of them were business women, 20% of them were career civil servants, 4.3% were skilled labourers/artisan, 4.8% students while the remaining 4.3% of them were unemployed. 81.3% of the participants gave birth in public hospitals, 11.3% of them gave birth in private hospitals while 7.4% gave birth at home. Majority of the participants (57.8%) had a monthly total family income of greater than N20, 000 (twenty thousand naira), (Table 1).

**Table 1:** Socio-demographic characteristics of participants

Demographic characteristic	Frequency (n= 230)	Percentage (%)
**Age Range (years)**		
< 20	21	9.1
20 - 30	126	54.8
31- 40	80	34.8
41- 50	1	0.4
> 50	2	0.9
**Marital status**		
Married	220	95.6
Separated/Divorce	3	1.3
Widow	0	0
Single	7	3.0
**Educational Qualification**		
No formal school	3	1.3
Primary school	13	5.7
Secondary school	113	49.1
Tertiary	101	43.9
**Occupation**		
Housewife	40	17.4
Business	112	48.7
Civil servants	46	20.0
Skilled labour/Artisan	10	4.3
Unemployed	10	4.3
Students	11	4.8
**Place of Delivery**		
Home	17	7.4
Govt. facility	187	81.3
Private facility	26	11.3
**Income (monthly) (N)**		
<20,000	97	42.2
21-50,000	68	29.6
51- 100,000	35	15.2
>100,000	30	13.0

### Knowledge about Exclusive Breastfeeding (EBF)

Most (86.5%) of the nursing mothers had a good knowledge and awareness about exclusive breastfeeding, 94.3% of the mothers were able to correctly define exclusive breastfeeding as a practice of feeding infants with breast milk only for the first 6 months of life. The advantages of breast milk over infant formula milk were well known to the mothers. The most frequently mentioned were its health benefits to the baby (59.1%), protection against infections (42.6%), right amount of nutrients and water (30%), ready availability (19.6%), cheapness (19.6%), and promotion of mother-child bonding (19.6%). When the mothers' knowledge of how long breast milk alone is adequate for the baby's nutritional requirement was tested, 60% of them indicated it was for 6 months, 26.1% indicated less than 6 months, 6.9% indicated more than 6 months, while another 6.9% were not sure. The majority (69.1%) of the mothers knew that breastfeeding should be initiated immediately after birth (within an hour), while 10% said it should be within 1- 24 hours and 17.4% of the mothers felt it should be dependent on the mother or baby's health. Most (78.3) of the mothers had received information about EBF from ante-natal clinics. Others got information from media (5.6%), friends/relations accounted for 2.2% of source of information, 11.3% claimed they got the information from all the sources, while 2.6% of the mothers said they have never received information about EBF from any of these sources (Table 2).

**Table 2:** Awareness and knowledge of the Participants about Exclusive Breastfeeding

Parameter	Frequency (N=230)	Percentage (%)
**Ever heard about EBF**		
Yes	199	86.5
No	31	13.5
**Definition of EBF**		
Correctly defined	217	94.3
Incorrectly defined	13	5.7
**Advantages of EBF**		
Makes baby healthier	136	59.1
Contains right amount of nutrients and water	69	30.0
Protect baby from infections	98	42.6
Readily available	45	19.6
Cheap	45	19.6
Strengthen mother-child bonding	45	19 .6
**Duration of EBF**		
<6 months	60	26 .1
6 months	138	60 .0
>6 months	16	6.95
Not sure	16	6.95
**Main source of information**		
Antenatal clinics	180	78.3
Media	13	5.6
Friends/Relation	5	2.2
Never heard of EBF	6	2.6
All of the above	26	11 .3
**Initiation of breastfeeding**		
Within an hour	159	69.1
Within 1- 24 hours	23	10
After given other fluids	6	2.6
After 24 hours of birth	1	0.4
Depends on mother or baby's health	40	17.4

Knowledge about EBF: Good (199= 86.5%)

### Practice of Exclusive Breastfeeding (EBF)

**Figure 1:**
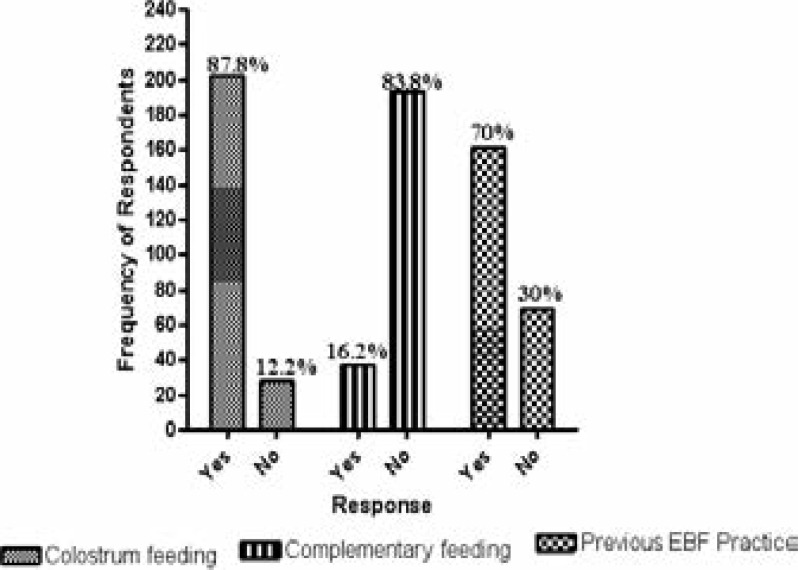
Feeding types by the Participants EBF= exclusive breastfeeding

Majority (83%) of the mothers indicated that they made their own decision to breastfeed while the remaining (17%) said they were influenced by their husband, mother/mother-in-law, sisters, friends and others. 65.7% of them made the decision before delivery while 34.3% was after delivery. About 85.2% of the participants admitted giving their babies' breast milk only immediately after delivery, while others gave water and herbs. 12.6% of them gave water and 1.3% gave herbs (Table 3). Colostrum feeding was highly practiced by the mothers (87.8%), and few (16.2%) mothers admitted giving their babies food or drink other than breast milk before six (6) months (figure 1). The majority (83.9%) of the mothers would breastfeed their baby any time baby demand for it. A high percentage of the mothers (70%) were practicing exclusive breastfeeding (figure 1).

**Table 3:** Practice of exclusive breastfeeding by the Participants

Parameter	Frequency (N=230)	Percentage (%)
**Decision on EBF**		
Self	191	83.0
Others	39	17.0
**Time of decision on EBF**		
Before birth	151	65.7
After birth	79	34.3
**Prelactal feeding**		
Breast milk only	196	85.2
Breast milk and water	29	12.6
Animal milk	2	0.9
Herbs	3	1.3
**Frequency of breastfeeding**		
On baby demand	193	83.9
When is convenient	22	9.6
Timed	15	6.5
**Duration of previous EBF**		
1-2 Months	18	7.8
3-4 Months	25	10.9
5 Months	15	6.5
6 Months	172	74.8

EBF= Exclusive breastfeeding, Practice of EBF = Good (70%)

### Barriers to the practice of Exclusive Breastfeeding (EBF)

The four most common barriers to the practice of the EBF from the study includes; insufficient milk production (46.1%), belief that infants require extra fluids (40%), fear of alteration in the breast figure or shape (39.6%) and cultural practice, societal beliefs (34.3%) and others (Table 4). As ways of ameliorating these barriers, the participants agreed on more public enlightenment campaigns among others solutions (Table 4).

**Table 4:** Barriers to the practice of EBF and solutions as agreed by the Participants

Barrier	Frequency (N=230)	Percentage (%)
Work status	56	24.3
Family members' influence	23	10.0

Mothers' health condition	67	29.1
Cultural Practice and Societal belief	79	34.3

Inadequate maternity leave and other work place policies	17	7.4
Inadequate knowledge on the benefits of EBF and proper breastfeeding techniques	26	11.3

Financial status of the family	15	6.6
Stress of education	10	4.3
Insufficient milk and lack of baby's satisfaction	106	46.1
Fear of breast sagging	91	39.6
Wrong believe that infants under six months require extra fluids in addition to breast milk especially in hot climate	92	40.0

**Solutions**		
Intensive EBF promotion efforts and educational activities	85	37.0
More Public enlightenment campaigns	92	40.0

Family support is required for successful practice of EBF	78	33.9
Employers of labour should grant waivers to nursing mothers to have time to breastfeed their infants	86	37.4
There should be more political commitment to all national policies on breastfeeding promotion	96	41.7
Maternity leave should be increased to 6 months	92	40.0

EBF= Exclusive breastfeeding

### Factors associated with initiation of Breastfeeding, Knowledge and Practice of EBF

Table 5a &5b illustrate the maternal factors associated with the practice of exclusive breastfeeding and the timely initiation of breastfeeding. Maternal age, educational qualification and occupation were significantly (p<0.05; p<0.001) associated with exclusive breastfeeding practice. Mothers aged 21-30 years, with higher educational status, with good occupation (business and civil servant) had significant association with the practice of exclusive breastfeeding. Mothers with higher educational qualification (secondary and above) were shown to have significant (p=0.004) association with the knowledgeable about exclusive breastfeeding (Table 5a). Similarly, maternal educational qualification and occupation were significantly (p=0.003; p=0.000) associated with initiation of breastfeeding.

**Table 5a:** Maternal factors associated with Exclusive Breastfeeding

Variable	Exclusive	Breastfeeding	X^2^	*p*-value
**Ages of mother**	**Yes**	**No**		
< 21	15(6.5)	9(3.9)	5.982	0.05*
21 – 30	50(21.7)	77(33.5)		
31 – 40	41(17.8)	38(16.5)		
41 – 50	-	-		
> 50	-	-		
**Mother's Qualification**				
Primary	31(14)	7(3.2)		
Secondary	60(27.1)	36(16.3)	14.748	0.001*
Tertiary	75(33.9)	12(5.4)		
**Mother's Occupation**				
Housewife	22(9.7)	17(7.5)		
Business	62(27.3)	34(15)	26.224	0.000*
Civil Servant	59(26)	3(1.3)		
Artisan	6(2.6)	3(1.3)		
Unemployed	6(2.6)	6(2.6)		
Student	6(2.6)	3(1.3)		
**Income (#)**				
<21,000	57(25.1)	38(16.7)		
21 – 50,000	48(21.1)	13(5.7)	3.995	0.262
50 – 100,000	16(7.0)	10(4.4)		
> 100,000	32(14.1)	13(5.7)		
**Marital Status**				
Married	179(77.8)	41(17.8)	0.910	0.634
Divorced	3(1.3)	-		
Single	6(2.6)	1(0.4)		

EBF= Exclusive breastfeeding

**Table 5b:** Maternal factors associated with Timely initiation of Breastfeeding

Variable	Timely Initiation of Breastfeeding		X^2^	*p*-value
**Ages of mother**	**Yes**	**No**		
< 21	15(6.4)	9(3.9)	1.766	0.414
21 – 30	85(36.5)	47(20.2)		
31 – 40	56(24)	21(9)		
41 – 50	-	-		
> 50	-	-		
**Mother's Qualification**				
Primary	5(2.3)	8(3.6)		
Secondary	70(31.5)	48(21.6)	11.421	0.003*
Tertiary	70(31.5)	21(9.5)		
**Mother's Occupation**				
Housewife	25(10.9)	17(7.4)		
Business	39(17)	56(24.3)	28.479	0.000*
Civil Servant	45(19.6)	17(7.4)		
Artisan	6(2.3)	3(1.3)		
Unemployed	3(1.3)	8(3.5)		
Student	11(4.8)	-		
**Income (#)**				
<21,000	54(23.6)	41(17.9)		
21 – 50,000	32(14)	32(14)	3.995	0.262
50 – 100,000	19(8.3)	6(2.6)		
> 100,000	29(12.7)	16(7)		
**Marital Status**				
Married	180(78.3)	40(17.4)	0.910	0.634
Divorced	2(0.9)	1(0.4)		
Single	5(2.2)	2(0.9)		

## Discussion

Our study indicated that the prevalence of exclusive breastfeeding practice in Kaduna metropolis was relatively high (70%) but more effort is still needed to meet the WHO recommendations of 90%. Most of the participants agreed that breastfeeding is ideal, protective, safe and nutritious for the child. The social-demographic characteristics of the participants are typical of women in urban cities in Nigeria, where high education and medium socio-economic status is very prevalent. Majority of the participants were business women, married, educated, had a total family income of more than twenty thousand naira and were still in their reproductive age. This study revealed that almost 99% of the participants had formal education unlike in a previous study in Yobe state where 59% of the participants had no formal education and only 24% had some form of formal education and 64% of the mothers are fulltime housewives 13. The level of education of the mothers in this study is similar to the results of studies in South-West Nigeria 1 14 where majority of the participants had high education.

The present study showed that there was high level of awareness and knowledge about EBF practice among mothers within Kaduna metropolis. This might be due to the fact majority of the mothers had access to media, over 92% of them gave birth in the hospital and were highly enlightened about the health benefits of EBF during the antenatal and postnatal clinics. This is very evident from the findings of this study, which showed that 78% of the participants got information about EBF from antenatal clinics. Also, 83% of them decided on their own to practice EBF. Mothers who attended antenatal clinics got appropriate messages about EBF and were more likely to practice it 4. Furthermore, majority of the participants agreed that exclusive breastfeeding has the advantages of making baby healthier, offers protection against infections and 60% of them correctly agreed that the duration of EBF should be six months as specified by WHO. This is higher than 44% obtained by 15 and similar to the 63.7% obtained in another study by 16. Our study showed that 59% of the participants agreed that exclusively breastfed children are protected against infections compared to the 66.7% obtained by 16.

Timely initiation of breastfeeding within an hour after delivery encourage the feeding of colostrum's to the infants. The present study showed that 69% of the participants correctly initiated breastfeeding within an hour after delivery. This is similar to 63.7% reported in a study conducted in Enugu, South-East Nigeria 16 and 63.4% reported in Tema Manhean, Ghana 17 but higher than 52.6% reported in Addis Ababa, Ethiopia 18 and 40.6% reported in South-West Nigeria 14. Colostrum was reported to offer protection against infections and unnecessary death 19. Pre-lacteal feeding is a common practice in Nigeria. However, in this study, only less than 15% of the nursing mothers gave pre-lacteal feeding in form of water, animal milk, herbs. The timely initiation of breastfeeding and low pre-lacteal feeding reported in this study can be attributed to the improved maternal education, understanding and appreciation of the benefits of colostrum 15.

Among the maternal factors associated with the exclusive breastfeeding, the age (p<0.05), educational qualification (p<0.001) and occupation (p<0.0001) of the nursing mothers were found to have significantly influence the exclusive breastfeeding rate in this study. The total monthly income of the family did not influence the initiation of breastfeeding and the practice of EBF. The mothers with secondary or higher education reported higher EBF rate in comparison to their counterparts with lower or no education. This is contrary to previous studies conducted in Sokoto 20, Sagamu 21 and in other studies reported by 1 22. However, similar findings were reported in Nigeria 4, in Enugu 16. There was an assumption that women with high level of educational qualification would have better breastfeeding practice due to better health education, knowledge and awareness about breastfeeding 10.

In this study, mothers between 21 and 30 years had association with the practice of EBF compared with others. This finding is consistent with the previous studies, which indicated that younger mothers were more likely to maintain EBF practice than older mothers 16,17, 22. This is probably because most mothers within this age range are more energetic, serious, educated and well informed of the benefits of breastfeeding to both infants and mothers 16. However, our finding is in contrast to the results of previous studies which indicated that maternal age had no association with the practice of EBF 1, 20, 23.

The study showed that mothers in business and civil service were shown to be associated with the practice of exclusive breastfeeding of their infants. This finding contradicts earlier reports which indicated that working mothers were less likely to exclusively breastfeed their infants 24. The mothers' occupation significantly (p<0.0001) influenced the initiation of breastfeeding as those doing business were more likely to initiate breastfeeding earlier than mothers engaged in other forms of occupation. This could be attribute to the fact that they knew that they were not going to be restricted by the official work schedules and can freely breastfeed their babies anytime they wish.

Results from our study revealed some of the barriers to the practice of EBF. The commonest ones are; insufficient milk production, cultural beliefs, the fear of alteration in the breast figure or shape, societal beliefs, stress of work place and the time-consuming nature of the EBF practice. This finding is supported by previous reports 1, 25,26. However, these can be overcome through the necessary support from family members, health care practitioners, government and all employers of labour as suggested by the participants. Adequate support from family, health workers and implementation of breastfeeding friendly work-related policies would create a comfortable and conducive environment required for the optimal practice of exclusive breastfeeding 27. It is therefore very commendable that the Kaduna state government has recently approved the extension of maternity leave from 3 months to 6 months for women who works in public service. This will go a long way in the support and promotion of EBF practice in the state to ensure survival, healthy growth and development of infants.

The results of this study are limited, findings of the study cannot be generalized for the whole Kaduna state as the study participants were recruited from modern health facilities in urban centre but could be considered indicative of the context considered. However, these findings revealed that the level of education attained by the breastfeeding mothers, availability of varied sources of information from care givers, family, friends, media etc. about the benefits of EBF greatly influenced the practice of EBF. We recommend more EBF interventions programs and workplace breastfeeding policies including extension of maternity leave to six months. Also, government and non-governmental organizations should intensify efforts at enlightenment campaign and practical education targeted at addressing factors associated with exclusive breastfeeding practice.

## Conclusions

Findings from this study showed that most of the participants had a very good knowledge of exclusive breastfeeding and are practicing it. Exclusive breastfeeding among mothers in Kaduna metropolis was influenced by mother's age, educational status and occupation.
